# Protein–protein interaction network–based evaluation of lung cancer relevance and hub gene identification from in vitro crocidolite exposure signatures

**DOI:** 10.1093/joccuh/uiag060

**Published:** 2026-09-26

**Authors:** Taisuke Tomonaga, Midori Iida, Hiroto Izumi, Shinya Morimoto, Yukiko Nakamura, Jun-ichi Takeshita, Toshiki Morimoto, Hidenori Higashi, Kazuhiro Yatera, Yasuo Morimoto, Chinatsu Nishida

**Affiliations:** Department of Occupational Pneumology, Institute of Industrial Ecological Sciences, University of Occupational and Environmental Health, 1-1 Iseigaoka, Yahata-nishi-ku, Kitakyushu, Fukuoka 807-8555, Japan; Department of Bioscience and Bioinformatics, Faculty of Computer Science and Systems Engineering, Kyushu Institute of Technology, 680-4 Kawazu, Iizuka-shi, Fukuoka 820-8502, Japan; Department of Occupational Pneumology, Institute of Industrial Ecological Sciences, University of Occupational and Environmental Health, 1-1 Iseigaoka, Yahata-nishi-ku, Kitakyushu, Fukuoka 807-8555, Japan; Department of Occupational Pneumology, Institute of Industrial Ecological Sciences, University of Occupational and Environmental Health, 1-1 Iseigaoka, Yahata-nishi-ku, Kitakyushu, Fukuoka 807-8555, Japan; Department of Occupational Pneumology, Institute of Industrial Ecological Sciences, University of Occupational and Environmental Health, 1-1 Iseigaoka, Yahata-nishi-ku, Kitakyushu, Fukuoka 807-8555, Japan; Research Institute of Science for Safety and Sustainability, National Institute of Advanced Industrial Science and Technology (AIST), 16-1 Onogawa, Tsukuba, Ibaraki 305-8569, Japan; Department of Respiratory Medicine, University of Occupational and Environmental Health, 1-1 Iseigaoka, Yahata-nishi-ku, Kitakyushu, Fukuoka 807-8555, Japan; Department of Environmental Health Engineering, Institute of Industrial Ecological Sciences, University of Occupational and Environmental Health, 1-1 Iseigaoka, Yahata-nishi-ku, Kitakyushu, Fukuoka 807-8555, Japan; Department of Respiratory Medicine, University of Occupational and Environmental Health, 1-1 Iseigaoka, Yahata-nishi-ku, Kitakyushu, Fukuoka 807-8555, Japan; Department of Occupational Pneumology, Institute of Industrial Ecological Sciences, University of Occupational and Environmental Health, 1-1 Iseigaoka, Yahata-nishi-ku, Kitakyushu, Fukuoka 807-8555, Japan; Department of Occupational Pneumology, Institute of Industrial Ecological Sciences, University of Occupational and Environmental Health, 1-1 Iseigaoka, Yahata-nishi-ku, Kitakyushu, Fukuoka 807-8555, Japan

**Keywords:** network analysis, protein–protein interaction network, crocidolite, hub gene, network propagation, lung cancer

## Abstract

**Objectives:**

This study evaluated whether a protein–protein interaction (PPI) network–based approach can link in vitro exposure-induced gene expression signatures to human disease modules.

**Methods:**

Crocidolite, a well-established lung carcinogen, was used as a model of inhaled dust. Human immortalized type I alveolar epithelial cells were exposed to 5 doses of crocidolite, and RNA sequencing was performed. We defined 253 asbestos exposure–responsive genes showing dose-dependent responses as a crocidolite module. Lung cancer–related proteins were compiled from OMIM, GWAS Catalog, PheWAS, and ClinVar, yielding a lung cancer module of 90 proteins. The network proximity between the crocidolite module and the disease modules was evaluated using a *z*-score. Hub genes associated with both crocidolite exposure and lung cancer were identified by cross-betweenness, Graph Kernel, and network propagation (NP). Method performance was assessed by area under the receiver operating characteristic curve (AUC) analysis using 11 guideline- and expert-selected lung cancer driver genes.

**Results:**

The crocidolite module showed significant proximity to the lung cancer module (Zc = −2.95), but not to the thyroid cancer module used as a negative control (Zc = 2.30). NP showed the highest performance for recovering driver genes (AUC = 0.974). NP-prioritized genes were enriched in phosphoinositide 3-kinase–protein kinase B (PI3K–Akt) signaling, cancer-related pathways, epidermal growth factor receptor/erythroblastic oncogene B (EGFR/ErbB) signaling, apoptosis, and cellular senescence. The top hub genes included *PARP1, STAT3, MYC, EGR1, CYP1A1*, and *SMAD3*.

**Conclusions:**

Integrating in vitro exposure transcriptomics with human disease modules through PPI-based network analysis may enable the computational evaluation of disease relevance and the identification of biologically meaningful hub genes for the assessment of occupational hazards.

## Teaser

**Key points**
 

- **What is already known on this topic:** Many chemicals are used in industrial sites, and efficient evaluation of their health effects is needed. Although animal exposure studies are important for toxicity assessment, alternative approaches are increasingly required. In vitro transcriptomics can capture cellular responses, but linking these responses to specific disease states remains challenging.
- **What this study adds:** This study shows that crocidolite-induced gene expression signatures from cultured alveolar epithelial cells can be linked to lung cancer–associated gene sets through protein–protein interaction network analysis. This approach evaluated network proximity between exposure-induced and disease-associated gene sets and identified hub genes that are potentially relevant to the development of lung cancer.
- **How this study might affect research, practice, or policy:** This framework may help to evaluate the disease relevance of molecular responses observed in in vitro studies and provide a basis for more efficient, mechanism-informed hazard assessment of inhaled particulate substances.

## 1. Introduction

With recent technological advancements, a wide variety of chemical substances are now used in industrial sites.^1^ Preventing health hazards to workers handling these chemicals is a crucial issue in industrial hygiene, and there is a need to efficiently and appropriately evaluate the health effects of chemical substances.^2^ Animal exposure testing is important for evaluating the toxicity of chemical substances and elucidating their pathogenesis, but in recent years there has been an international demand to reduce animal testing from the perspective of animal welfare and ethics.^3^^,^^4^ Therefore, there is a need to develop new approaches to elucidating the pathogenesis of chemical substances and to evaluating their toxicity based on their pathogenesis, as an alternative to animal testing.^3^^,^^4^

Among these new approaches, cell culture testing is a promising method for evaluating the toxicity of chemical substances, but it has traditionally been limited in its use for predicting pathogenesis because it cannot reproduce the pathogenesis seen in animal testing. On the other hand, pathogenesis results from changes in the expression of numerous genes, and comprehensive gene expression analysis can be performed in cell culture testing. Therefore, if gene expression signatures associated with pathogenesis can be extracted from cell culture assays, this approach is expected to provide important toxicity assessment data that can be used in the hazard assessment of inhaled substances.

Originally, the cellular response to chemical stimulation, which is the endpoint of cultured cell studies, involved gene expression that led to disease states as well as gene expression that maintained homeostasis. As a result, it has been difficult to determine which specific gene expression changes were associated with which disease state. However, as gene expression data associated with human disease states have become increasingly available, it is now possible to compare exposure-induced gene expression signatures from cultured cells against disease-associated gene sets, thereby extracting signatures potentially implicated in disease development. For example, gene sets with altered expression related to lung cancer can now be obtained from publicly available datasets. Furthermore, individual genes in these gene expression groups do not act in isolation but also influence the expression of multiple other genes. That is, many gene expressions fluctuate through a network of interactions (a graphical structure with genes as nodes and intergenic interactions as edges) consisting of close signal transduction and binding between genes. In particular, genes that occupy central positions within a network and play crucial roles in disease initiation and progression are referred to as “hub genes,” some of which may function as driver genes. Altered expression of these genes is closely associated with disease states. Network-based approaches have been used to identify disease-relevant hub genes in various cancers; for example, competing endogenous RNA (ceRNA) network analysis has been applied to identify prognostic hub genes in stomach adenocarcinoma.^5^

In this study, we applied a protein–protein interaction (PPI) network–based approach to evaluate the association between exposure-induced gene expression profiles from cultured cells and disease-associated gene sets compiled from public databases.

As a proof-of-concept analysis, we used crocidolite asbestos and lung cancer–associated gene sets. Crocidolite was selected because this study focused on inhaled occupational dusts rather than chemical lung carcinogens such as nitrosamine 4-(methylnitrosamino)-1-(3-pyridyl)-1-butanone (NNK). Although chronic inflammation is a hallmark of asbestos-related carcinogenesis,^6^ the present study did not aim to reproduce the entire carcinogenetic process in vitro. Instead, we quantitatively evaluated the network proximity between crocidolite-induced gene sets and lung cancer–associated gene sets and qualitatively assessed whether hub genes potentially relevant to lung cancer initiation and progression could be identified within the shared network region.

## 2. Materials and methods

### 2.1 Sample preparation

The crocidolite [Na_2_Fe_3_^2+^Fe_2_^3+^(Si_8_O_22_) (OH)_2_] used in this study was Union Internationale Contre le Cancer (UICC) asbestos. The crocidolite preparation, measured by scanning electron microscopy, had a geometric mean diameter of 0.2 μm (geometric standard deviation [GSD] 1.5) and a geometric mean length of 1.3 μm (GSD 2.3).^7^ A water suspension of crocidolite (2.5 mg/mL) was used in the experiments.

### 2.2 Experimental data and asbestos exposure–responsive genes

To identify genes responsive to crocidolite exposure, human immortalized type I alveolar epithelial cells (T0487; Applied Biological Materials Inc) were seeded in 60-mm dishes at 5 × 10^5^ cells/dish in 3 mL of culture medium. After 24 hours, cells were exposed to crocidolite at 0, 1, 2.5, 5, or 10 μg/mL (N = 1 culture per concentration). After 72 hours, total RNA was isolated using the RNeasy Mini Kit (Qiagen, Hilden, Germany), and gene expression profiles were measured by RNA sequencing and quantified as transcripts per million (TPM). Asbestos exposure–responsive genes were defined by the following criteria: (1) a coefficient of determination (*R*^2^) ≥0.6 for the linear dose–response relationship; (2) |log_2_ fold change >1.2; (3) minimum expression level of TPM ≥2; and (4) *P* value <0.05 for the dose–response correlation. Genes meeting all 4 criteria were defined as asbestos exposure–responsive genes, yielding 253 genes (crocidolite module).

### 2.3 Disease-related genes

Lung cancer–related genes (lung cancer module) and thyroid cancer–related genes (thyroid cancer module) were compiled from 4 databases: OMIM, the GWAS Catalog, PheWAS, and ClinVar. After duplicate entries were removed, 90 and 174 genes were retained for the lung cancer and thyroid cancer modules, respectively.

### 2.4 Protein–protein interaction (PPI) network

Human PPI data were obtained from 10 databases, including APID, BioGRID, CORUM, HI-II-14, HuRI, InnateDB, Instruct, KEGG rpair, PhosphoSitePlus v6.6.0.4, and Signalink v3.0, as well as previously reported data.^8^ The integrated PPI network comprised 437 922 interactions among 18 570 genes encoding proteins (mean degree = 47.16). The largest connected component was extracted, retaining 437 920 interactions from 18 566 genes.

### 2.5 Network proximity analysis

Network proximity between the crocidolite module and disease modules was quantified using a *z*-score (Zc) based on the method described by Guney et al.^9^ For 2 gene modules, S (disease module) and T (crocidolite module), the proximity d(S,T) was defined as the mean shortest path distance from each node in T to its nearest node in S within the PPI network. Statistical significance was assessed by a degree-preserving permutation test (1000 iterations), in which random gene sets of the same size as the original modules were sampled from nodes with similar network degrees, generating a null distribution with mean μ and SD σ. The Zc was calculated as:


$${Z}_c=\frac{\left(d\left(S,T\right)-\mu \right)}{\sigma }$$

A negative Zc value indicates greater co-localization of the 2 modules in the PPI network than expected by chance.

### 2.6 Hub gene identification by network-based methods

To identify hub genes associated with both crocidolite exposure and lung cancer, 3 network-based methods were applied to the PPI network using the crocidolite module and the lung cancer module as inputs:

1. Cross-betweenness (CB), a distance-based metric that quantifies the extent to which nodes lie on the shortest path connecting the crocidolite module and the lung cancer module.

2. Graph Kernel (GK), a network diffusion method implemented using the diffStats R package. Scores were independently propagated from the crocidolite module and the lung cancer module across the PPI network using a regularized Laplacian kernel,^10^ and the final score for each node was computed as the product of the 2 resulting score vectors.

3. Network propagation (NP),^11^ a diffusion-based method that independently spreads signals from each gene of the crocidolite and lung cancer modules across the PPI network using random walk with restart, and computes the final node score as the product of the 2 propagated scores.

For all 3 methods, each node in the PPI network was assigned a score reflecting its association with both crocidolite exposure and lung cancer. The top 200 nodes with the highest scores were defined as hub genes for each method.

### 2.7 Enrichment analysis

Kyoto Encyclopedia of Genes and Genomes (KEGG) pathway enrichment analysis was performed to estimate the signaling pathways and biological functions involved in the top 200 genes extracted by NP.

### 2.8 Performance evaluation of the 3 network-based methods

Receiver operating characteristic (ROC) curve analysis was performed to investigate the hub gene identification performance of the methods. A set of 11 gene symbols corresponding to clinically actionable non-small cell lung cancer (NSCLC) driver alteration categories was selected based on clinical guidelines and expert judgment and used as the positive label set (*EGFR, KRAS, ALK, ROS1, BRAF, MET, RET, NTRK1, NTRK2, NTRK3, HER2*). These genes correspond to established actionable driver categories in non-small-cell lung cancer. For each method, nodes in the PPI network were ranked by their association scores, and the ability to recover the positive label set was evaluated by computing the area under the ROC curve (AUC).

To further evaluate the robustness of method performance, ROC analysis was repeated using an independent IntOGen-derived lung cancer driver gene set. IntOGen is a public resource of mutational cancer driver genes identified by integrating multiple computational driver-discovery methods.^12^ lung adenocarcinoma (LUAD) and lung squamous cell carcinoma (LUSC) driver gene sets were downloaded from the IntOGen database (https://www.intogen.org/download; accessed August 12, 2026), and duplicate gene symbols were removed, yielding 128 unique genes. These genes were used as an additional lung cancer–specific benchmark set.

## 3. Results

### 3.1 Network proximity between crocidolite and disease modules

To quantitatively assess whether crocidolite exposure shares network-level pathological mechanisms with lung cancer, network proximity was calculated as a *z*-score (Zc) between the crocidolite module and 2 disease modules: lung cancer and thyroid cancer. Thyroid cancer was selected as a negative control, as it has no established causal relationship with asbestos exposure. The Zc value indicates how close 2 gene modules are in the PPI network relative to random expectation; a more negative value indicates greater network proximity. The Zc between the crocidolite module and the lung cancer module was −2.95, whereas the Zc between the crocidolite module and the thyroid cancer module was +2.30 (Figure 1a, 1b). These results demonstrate that the crocidolite module is significantly co-localized with the lung cancer module in the PPI network, whereas no such co-localization was observed with the thyroid cancer module.

**Figure 1:**
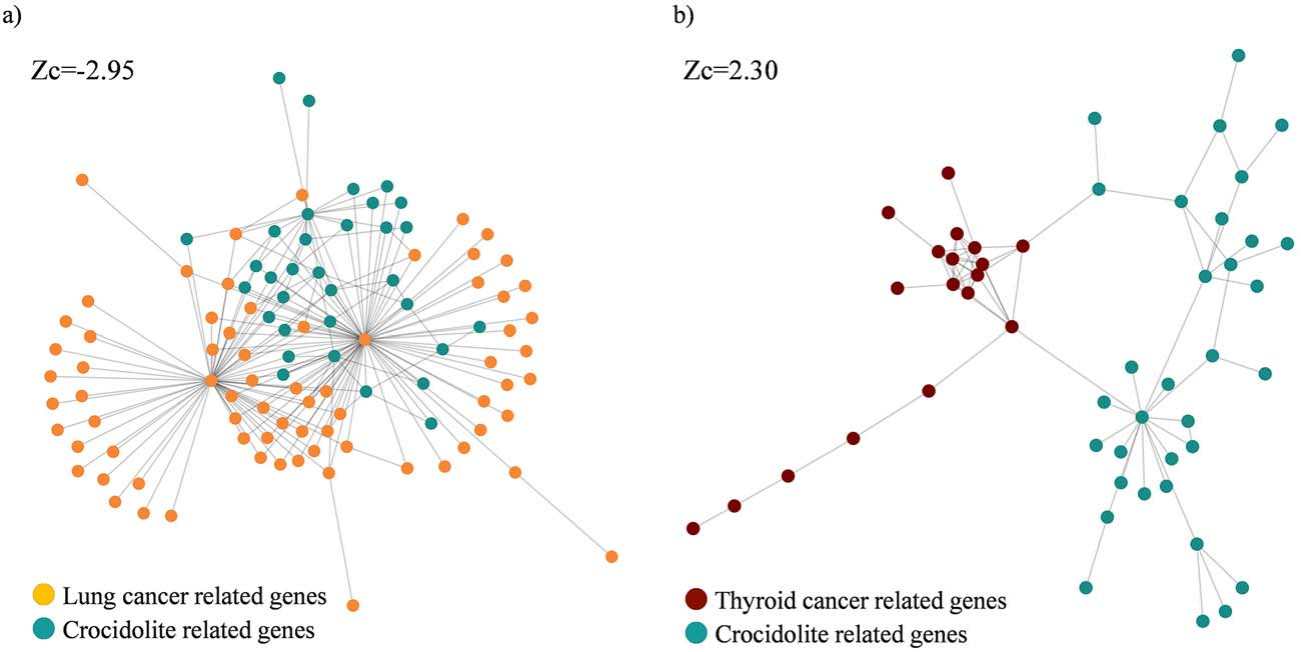
Network proximity between the crocidolite module and disease modules. Subgraph visualization of the protein–protein interaction (PPI) network showing the overlap between the crocidolite module and (a) the lung cancer module (Zc = −2.95) and (b) the thyroid cancer module (Zc = +2.30). The *z*-score (Zc) represents network proximity relative to random expectation; a more negative value indicates greater co-localization in the PPI network.

### 3.2 Enrichment analysis of NP-derived genes

KEGG pathway enrichment analysis of the genes prioritized by NP showed that the phosphoinositide 3-kinase–protein kinase B (PI3K–Akt) signaling pathway was highly ranked (Figure 2a). Cancer-related pathways, epidermal growth factor receptor/erythroblastic oncogene B (EGFR/ErbB)-related signaling pathways, apoptosis, and cellular senescence–related pathways were also significantly enriched.

**Figure 2:**
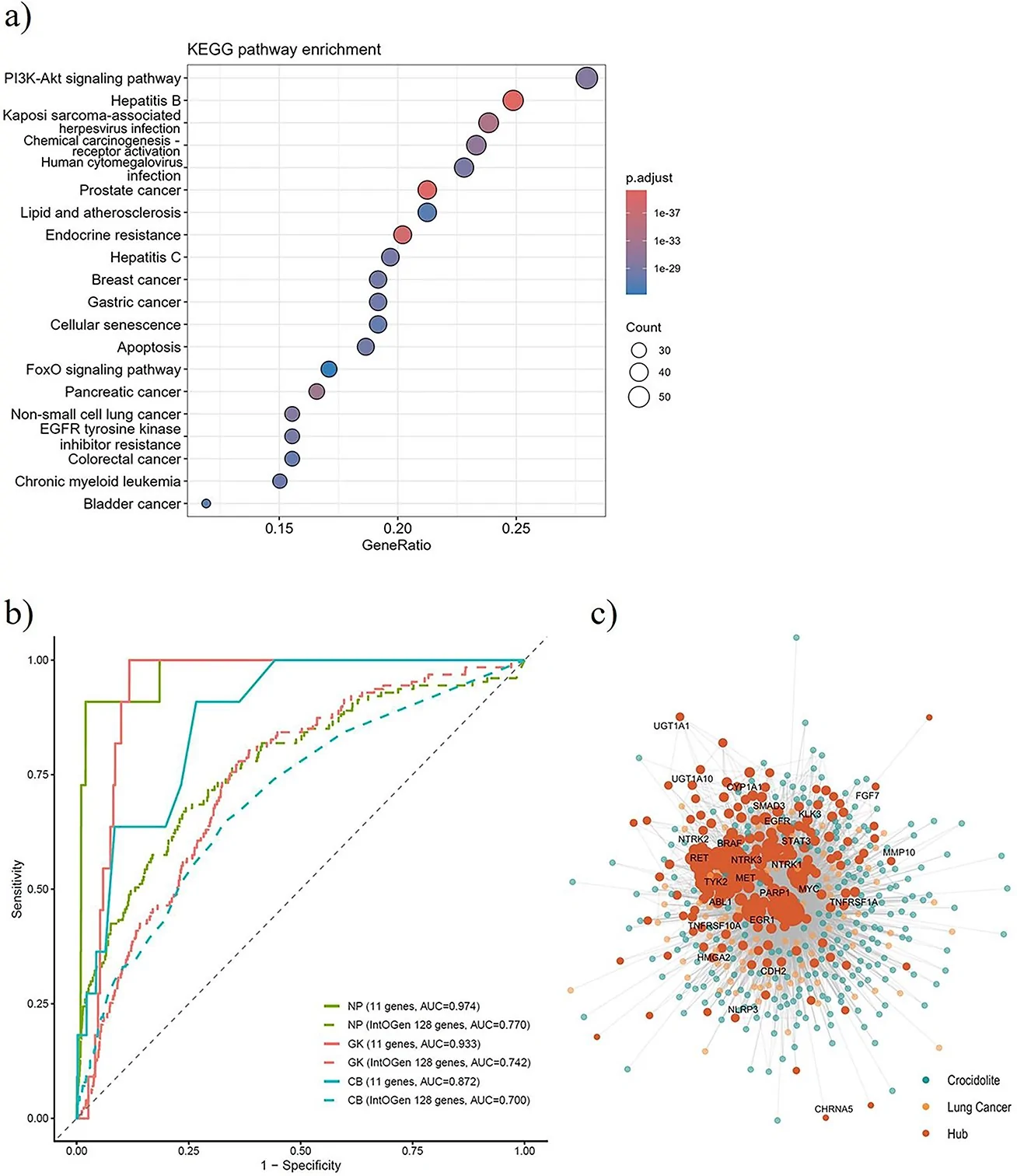
(a) KEGG pathway enrichment analysis of network propagation-derived genes. (b) Receiver-operating curve (ROC) analysis comparing cross-betweenness (CB), graph kernel (GK), and network propagation (NP) using 11 guideline- and expert-selected lung cancer driver genes and an IntOGen-derived 128-gene lung cancer driver set as benchmark sets. (c) PPI network structure of the hub genes identified by NP alongside crocidolite exposure–responsive genes (crocidolite module) and lung cancer-related proteins (lung cancer module).

### 3.3 Performance evaluation of hub gene identification methods

The performance of the 3 network-based methods was evaluated by ROC curve analysis using 11 guideline- and expert-selected lung cancer driver genes as the positive label set (Figure 2b). NP achieved the highest AUC (0.974), followed by GK (0.933) and CB (0.872), indicating that, among the 3 methods, NP prioritized the known lung cancer driver genes the most effectively. Although the AUC values were lower in this broader benchmark set, NP again showed the highest AUC (0.770), followed by GK (0.742) and CB (0.700), supporting the robustness of NP as the best-performing method among the 3 methods.

### 3.4 Hub genes identified by the NP method

Based on the NP analysis, genes with high association scores reflecting proximity to both the crocidolite module and the lung cancer module in the PPI network were extracted from their shared network region, and the top 200 were selected as hub genes for downstream analysis. The top 10 hub genes ranked by association score included *PARP1, STAT3, MYC, EGR1, CYP1A1, SMAD3, NLRP3, AR, HMGA2*, and *ABL1*. Among these genes, *PARP1* (hazard ratio [HR] = 1.9, *P* = .01) and *HMGA2* (HR = 1.9, *P* = .011) showed significant associations with worse overall survival in The Cancer Genome Atlas (TCGA) mesothelioma patients, an asbestos-related malignancy (Figure S1). Of the 11 guideline- and expert-selected lung cancer driver genes used as the positive label set for ROC evaluation, 7 were identified among the hub genes: *EGFR, BRAF, MET, NTRK1, NTRK2, NTRK3*, and *RET* (Figure 2c). These findings suggest that crocidolite exposure activates gene networks that overlap with established lung cancer driver pathways.

## 4. Discussion

This study evaluated the network-based proximity between crocidolite exposure–associated gene expression signatures obtained from human immortalized type I alveolar epithelial cells and lung cancer–associated gene sets derived from publicly available human disease databases. Furthermore, we extracted subnetworks and hub genes located in the vicinity of both modules using NP. To examine whether the gene expression signatures derived from alveolar epithelial cells are valid as crocidolite-induced oncogenesis-related signatures, we considered the following 3 points: (1) evaluation of the network-based proximity between the crocidolite exposure module and the lung cancer module; (2) gene prioritization patterns in the shared network region and hub genes identified by NP; and (3) the validity of selecting the NP method used in this study.

*1. Evaluation of the network-based proximity between the crocidolite exposure module and the lung cancer module.* The Zc score, which evaluates the network-based proximity between modules, showed that the Zc between the crocidolite exposure module and the lung cancer module was −2.95, whereas the Zc between the crocidolite exposure module and the thyroid cancer module was +2.30. Calculation of the Zc value is based on the shortest path lengths between genes contained in each module. A larger negative value indicates greater network-based proximity between modules, whereas a larger positive value indicates greater separation between the 2 modules within the network. Given that crocidolite is a carcinogen with an epidemiologically established association with lung cancer,^13^ the results of this study suggest that this network analysis may appropriately reflect the association between crocidolite exposure and lung cancer, supporting the usefulness of this method. This evaluation method is also used in approaches to the discovery of drugs, including the prediction of whether disease modules and drug modules are closely located within a network; that is, whether a drug may be effective against a disease. In type 2 diabetes, for example, the Zc value for the hypoglycemic agent gliclazide was reported to be −3.3.^9^ As shown in the present study, a similar framework may be applicable for evaluating associations between chemical substances and diseases.

*2. Gene prioritization patterns in the shared network region and hub genes identified by network propagation.* No significant pathways were detected in the enrichment analysis of the crocidolite-induced asbestos exposure–responsive genes (data not shown). On the other hand, by applying NP, genes located in the neighborhood of both the crocidolite exposure module and the human lung cancer module were preferentially prioritized according to their network-based proximity. This approach allowed the extraction of genes involved in cell proliferation and survival signaling, carcinogenesis-related pathways, and apoptosis. In particular, the PI3K–Akt signaling pathway is considered an important survival signaling pathway that regulates various processes related to cancer malignancy, including not only cell proliferation but also suppression of apoptosis, invasion and metastasis, and drug resistance.^14^ These findings suggest that gene expression profiles obtained from cultured cell studies show molecular features closely associated with lung cancer–related networks.

Next, the validity of the network analysis was evaluated based on the extracted hub genes. Based solely on the gene expression signatures obtained from cultured cell studies, only 2 hub genes overlapped with lung cancer–associated genes. However, considering the network-based proximity between the 2 modules, the association between crocidolite exposure and lung cancer was suggested to be formed not by the simple overlap of genes, but through indirect interactions within the human molecular interaction network. Therefore, after NP, approximately 18 000 genes in the network were scored, and genes with high proximity to both modules were prioritized as candidate hub genes. In particular, changes in the expression of driver genes functionally related to the initiation and progression of lung cancer were observed. Furthermore, among the top-ranked genes, *MYC, NTRK1*, and *SMARCA4* have been reported to be associated with lung cancer, and their involvement in the regulation of lung cancer development, progression, differentiation, and proliferation has been suggested.^15^ Additional survival analysis of the top-ranked hub genes showed that high expression of *PARP1* and *HMGA2* was associated with worse overall survival in The Cancer Genome Atlas Mesothelioma (TCGA-MESO) dataset. Although this analysis provided complementary clinical support for the relevance of *PARP1* and *HMGA2* in an asbestos-related malignancy, TCGA-MESO is not a cell type– or tissue-matched validation cohort and does not directly validate hub gene activation in asbestos-induced lung cancer. Further validation using independent asbestos-related lung cancer cohorts and experimental assays, including quantitative polymerase chain reaction (qPCR) and protein-level analyses, will be required. In this study, the ability to extract lung cancer–related hub genes from crocidolite exposure signatures, using crocidolite as a known inhaled carcinogen,^13^ supports the potential usefulness of this method for evaluating associations between chemical exposure and disease.

*3. Comparison of hub gene extraction methods and selection of NP in this study.* In this study, 3 methods were compared for extracting hub genes: CB, GK, and NP. The CB method prioritizes nodes located on the shortest paths connecting the crocidolite exposure module and the lung cancer module. While the CB method is a shortest path–based method suitable for detecting direct bridging genes between the 2 modules, the GK method and the NP method are both diffusion-based methods, based on the algorithms of Vandin et al^10^ and Vanunu et al,^11^ respectively. Both methods propagate scores from the crocidolite exposure–associated gene set and the lung cancer–associated gene set to neighbor nodes within the network in a ripple-like manner. In this study, each gene was evaluated by the product of the crocidolite exposure–related score and the lung cancer–related score obtained from each method.

Performance evaluation using ROC curve analysis showed that the NP method achieved the highest performance, with an AUC of 0.974, compared with the CB method (AUC 0.872) and the GK method (AUC 0.933). Because the initial ROC analysis used a small set of clinically actionable NSCLC driver genes, the AUC may overestimate the overall prioritization performance. However, NP again showed the highest AUC in an additional benchmark analysis using an IntOGen-derived 128-gene lung cancer driver set, supporting the robustness of NP as the best-performing method in the present framework. Although all 3 methods showed relatively consistent performance, the NP method was selected for this study because it demonstrated the best performance.

A key limitation of this study is that the RNAseq experiment lacked biological replicates; therefore, the asbestos exposure–responsive genes should be interpreted as exploratory dose-responsive seed genes.

## 5. Conclusions

In conclusion, this study demonstrated that PPI-based network analysis integrating in vitro chemical exposure data with external disease databases can computationally evaluate associations between chemical exposure and human disease and extract cancer-related hub genes. The extracted hub genes may provide important clues for elucidating the pathogenesis of diseases caused by chemical exposure and for identifying disease biomarkers. This method is expected to be applicable to other chemicals and diseases by selecting appropriate disease databases and cell models.

## Supplementary Material

Supplementary_materials_uiag060

## Data Availability

The protein–protein interaction data used in this study were obtained from publicly available databases. The experimental datasets generated and/or analyzed during the current study are not publicly available because they form part of ongoing research, but they are available from the corresponding author upon reasonable request. Details of the analytical methods and statistical programming code are also available upon reasonable request.
