# Supplementary material for: Protein–protein interaction network–based evaluation of lung cancer relevance and hub gene identification from in vitro crocidolite exposure signatures
Source: J Occup Health. 2026 Sep 26;68(1):uiag060. doi: 10.1093/joccuh/uiag060 (PMC13755352; doi:10.1093/joccuh/uiag060)
Supplement: Supplementary file 1 [file supplementary_materials_uiag060.docx]

**Supplementary materials**

This appendix has been provided by the authors to give readers additional information about their work.

Supplement to: Taisuke Tomonaga, Midori Iida, Hiroto Izumi, et al. Protein–protein interaction network-based evaluation of lung cancer relevance and hub gene identification from in vitro crocidolite exposure signatures

**Supplemental Methods**

**Complementary clinical assessment using the TCGA mesothelioma dataset**

To evaluate the clinical relevance of the identified hub genes in an asbestos-related malignancy, overall survival (OS) analysis was performed using The Cancer Genome Atlas (TCGA) mesothelioma dataset (MESO; n = 82), accessed via GEPIA2 (http://gepia2.cancer-pku.cn/; accessed July 29, 2026). Mesothelioma was selected because it is strongly associated with asbestos exposure. Patients were stratified into high and low expression groups based on the median expression cutoff (n = 41 per group). Differences in OS between groups were assessed using the log-rank test, and hazard ratios (HR) with corresponding p-values were calculated.

**Figure S1. Overall survival analysis of hub genes in TCGA mesothelioma (MESO) dataset.**

Kaplan–Meier survival curves for the top ten hub genes identified by network propagation analysis are shown using the TCGA MESO dataset (n = 82; n = 41 per group), accessed via GEPIA2 (http://gepia2.cancer-pku.cn/; accessed July 29, 2026). Patients were stratified into high (red) and low (blue) expression groups based on the median expression cutoff. Hazard ratios (HR) and log-rank p-values are indicated in each panel. Mesothelioma was selected as an asbestos-related malignancy to provide a complementary clinical assessment in the context of asbestos exposure. Among the ten hub genes, PARP1 (HR = 1.9, p = 0.01) and HMGA2 (HR = 1.9, p = 0.011) showed statistically significant associations with worse overall survival, suggesting the potential clinical relevance of these network-identified genes in an asbestos-related malignancy.
